# New Light on an Old Story: Lymphocystis Disease in Copperband Butterflyfish (*Chelmon rostratus*) and Orbicular Batfish (*Platax orbicularis*)

**DOI:** 10.3390/pathogens14100988

**Published:** 2025-10-01

**Authors:** Márton Hoitsy, Endre Sós, János Gál, Árisz Ziszisz, Ferenc Baska, Lars August Folkman, Giuseppe Mark Marcello, Krisztina Bali, Gergő Mitró, Andor Doszpoly

**Affiliations:** 1 Department of Exotic Animal, Wildlife, Fish and Honeybee Medicine, University of Veterinary Medicine Budapest, H-1078 Budapest, Hungary; drsos.endre@zoobudapest.com (E.S.); gal.janos@univet.hu (J.G.); ziszisz.arisz@univet.hu (Á.Z.); baska.ferenc@univet.hu (F.B.); 2 Fish Diseases Research Group, Division of Fish Medicine and Aquaculture, Department of Exotic Animal, Wildlife, Fish and Honeybee Medicine, University of Veterinary Medicine Budapest, H-1078 Budapest, Hungary; 3 Budapest Zoo and Botanical Garden, H-1146 Budapest, Hungary; 4 Vet4Fish Ltd., H-2111 Szada, Hungary; 5 Department of Production Animal Clinical Sciences, Faculty of Veterinary Medicine, Norwegian University of Life Science, 4325 Sandnes, Norway; lars.august.folkman@nmbu.no; 6 Vet Partners Ltd., LLM Farm Vets Derbyshire, Bakewell DE45 1AH, UK; mark.marcello@llmvets.co.uk; 7 Department of Microbiology and Infectious Diseases, University of Veterinary Medicine Budapest, H-1078 Budapest, Hungary; krisz.bali@gmail.com; 8 HUN-REN Veterinary Medical Research Institute, H-1143 Budapest, Hungary; mitro.gergo@vmri.hun-ren.hu (G.M.); doszpoly.andor@vmri.hun-ren.hu (A.D.)

**Keywords:** marine fish, lymphocystis, Iridoviridae, batfish, butterflyfish

## Abstract

Although the clinical course and pathogenesis of lymphocystis disease virus (LCDV) infection have been extensively described in freshwater and seawater environments, lymphocystis disease has not been studied in the copperband butterflyfish (*Chelmon rostratus*) or described at the molecular level in orbicular batfish (*Platax orbicularis*). The present study aimed to identify LCDV in a copperband butterflyfish and an orbicular batfish using light and electron microscopy (morphological) and molecular methods, namely PCR followed by phylogenetic analysis. We present a case series of two representatives of two distinct fish species with stress-induced chronic LCDV infection, which presented with typical, recurring, macroscopically visible lymphocystis nodules on their pectoral, caudal, and dorsal fins. After collecting lymphocystis nodules from live animals using skin scraping, we processed the hypertrophic giant cells for qualitative analysis using light and electron microscopy. Through our qualitative morphological analysis, we also share intimate observations of putative viral replication and assembly in the intracytoplasmic inclusion bodies of lymphocystis nodules. We present LCDV infection in a novel species, the copperband butterflyfish, and our molecular analysis identified the virus from the orbicular batfish as a novel LCDV species.

## 1. Introduction

With the sustained popularity of marine aquariums, thousands of marine fish species are traded worldwide. The ornamental fish business has an estimated annual value of between $800 million and $30 billion [1,2,3]. Marine aquariums are more difficult to maintain than freshwater aquariums. The filtration system is more complex, and it is more difficult to maintain appropriate water parameters in a marine system. Water quality, filtration, and tank community play important roles in the health status of animals [4,5]. Small differences in water quality can cause stress or serious health problems in several fish species [5].

The copperband butterflyfish (*Chelmon rostratus*, Linnaeus, 1758) is a common pelagic fish found along coral reefs and on rocky shores. It has a silvery-white body adorned with three wide vertical bars that range from orange to copper and reach a maximum length of 20 cm [4,6]. The head begins with a characteristic long snout adorned with a thinner copper band that runs through the eye. The animals are monogamous and form pairs during the breeding season, but are territorial and therefore should be kept as singles. Butterflyfish usually feed on benthic invertebrates [6,7].

Orbicular batfish *(Platax orbicularis*, Forsskål, 1775) are large batfish species that live mostly in the pelagic zone of coral reefs and mangrove habitats, feeding on invertebrates, small fish, and algae [6,8]. Orbicular batfish lay pelagic eggs [9]. Juvenile fish usually live alone or in small groups. They are reddish-brown with elongated vertical fins and a black bar running through their eyes. They mimic plant debris in shallow water, mangrove forests, and near the surface [8]. Adult batfish live in groups in deeper waters and have silver bodies with dark, broad, vertical bands that reach 40 cm in length [8,10].

Viral infections have been shown to cause high mortality rates in fish, both in freshwater and marine aquaculture, as well as in ornamental fish aquaria [5,11,12,13,14,15,16]. Lymphocystis disease (LCD) is a common viral infection in ornamental fish. Lymphocystis disease viruses (LCDV) are classified into the genus *Lymphocystivirus*, belonging to the family Iridoviridae [5,17,18]. According to the literature, more than 150 fish species, including freshwater and marine fish species, can be affected by LCDV [17,19]. LCDV in fish is a self-limiting, chronic disease characterised by white, nodular growths on the external body surface and fins. The virus replicates in the dermal fibroblast cells, resulting in impressive hypertrophy of these cells, increasing up to 50–100 times larger than normal [20,21]. The virus spreads when these giant infected fibroblast cells rupture, releasing virions into the environment. The incubation period of LCDV ranges from several weeks to months; therefore, infected fish may exhibit a delayed onset of clinical signs [5].

Despite the low morbidity and mortality rates of LCDV infection, the disease has economic and clinical significance. LCDV in the finfish aquaculture industry has the potential to decrease the commercial value of fish [22]. Additionally, in aquaria collections, clearly visible lymphocysts on ornamental fish provide a clinical indication of underlying immune compromise, which may inform individual and group management decisions [21,23].

Stress plays an important role in the epidemiology of LCD [24]. There are a myriad of perceived stressors for ornamental fish in aquariums, such as suboptimal water quality, overpopulation, predators in the same exhibit, inadequate nutrition, increased parasite burden, and transport. LCD may be observed subsequent to any number of these stressors acting alone or compounding these effects. It is no surprise that lymphocysts may be observed following transport, as this major stress event significantly disturbs the physical and chemical environment of fish for an extended period of time, sometimes more than 24 h [25,26]. Transport may be seen as a significant test of the resilience of the immune system of individual fish, and transport itself may be an immunocompromising event, which may result in the manifestation of LCD [26,27]. Following immunocompromise secondary to stress events, LCD may inform management decisions, motivating keepers to mitigate unavoidable stress events, such as transport.

In the present study, we aimed to identify the pathogens that caused LCD-like clinical signs in copperband butterflyfish and orbicular batfish using molecular, histological, and electron microscopic methods.

## 2. Materials and Methods

### 2.1. Sample Collection and Histology

The animals arrived at the marine aquarist shop after 23 h of transport, where they were placed into the quarantine system (T: 25.2 °C, salinity: 1.026, KH: 6.5, NO_3_^−^: 20 ppm, Ca: 460 ppm, Mg: 1410 ppm). Clinical signs manifested 3 days after quarantine housing. Tissue samples were collected from the external surfaces of both fish by skin scraping while under anaesthesia. Both fish were anesthetized with 2-Phenoxyethanol (0.2 mL/l, ≥99%, Thermo Fisher Scientific Inc., Waltham, MA, USA).

A Canon 250D (Canon Inc., Tokyo, Japan) DSLR camera with a Sigma 105 mm F2.8 DG DN macro objective (Sigma Corporation, Tokyo, Japan) was used to capture macroscopic images. Native cytological samples were collected from the affected areas of the body with visible clinical signs, including the pectoral, dorsal, and caudal fins. Native tissues were examined using a Euromex BioBlue BB.4260 microscope (Euromex Microscopen BV, Duiven, The Netherlands). The samples were fixed in 10% neutral-buffered formalin for 24 h. Following fixation, the tissues were embedded in paraffin. Histological sections (3 μm slices) were prepared and stained with haematoxylin and eosin (H&E) from tissue blocks. The slides were investigated using a Euromex BioBlue BB.4260 microscope and digitised using a Panoramic MIDI II (3DHistech Kft, Budapest, Hungary) scanner. Histopathological images were prepared using CaseViewer (3DHistech Kft, Budapest, Hungary).

### 2.2. PCR Assay

Total DNA was extracted from one of the nodular lesions of the orbicular batfish using the NucleoSpin DNA RapidLyser kit (Macherey-Nagel, Dueren, Germany). The first PCRs targeted the DNA polymerase (DNA pol) and major capsid protein (MCP) genes of LCDV. After the successful identification of LCDV in the sample, additional core genes (DNA-dependent RNA polymerase largest subunit (RNA pol), ATPase, deoxynucleoside kinase, and a hypothetical protein [core gene 20]) were also amplified. The primers and reaction mixtures were described by Kitamura et al. and Kvitt et al. [28,29] for the DNA pol and MCP genes, and by Perretta and coworkers [30]. PCR products were purified using the NucleoSpin Gel and PCR Clean-up kit (Macherey-Nagel, Düren, Germany) after being excised from agarose gel (1%). Sequencing reactions were performed using the BigDye Terminator v3.1 Cycle Sequencing Kit (Applied Biosystems, Waltham, MA, USA) with forward and reverse primers. Subsequently, electrophoresis was performed by a commercial service provider using an ABI PRISM 3100 Genetic Analyser (Thermo Fisher Scientific Inc., Waltham, MA, USA).

For DNA extraction from paraffin-embedded tissues originating from copperband butterflyfish, the NucleoSpin DNA RapidLyser kit was used after xylene treatment, as described in detail by Pikor and coworkers [31]. DNA concentrations were measured using a Qubit 4 Fluorometer (ThermoFisher Scientific, Waltham, MA, USA). Non-specific amplification of the total DNA was performed using the Repli-g Mini Kit (Qiagen, Hilden, Germany).

### 2.3. Phylogenetic Analysis

Sequence handling and analyses were described in detail by Perretta et al. [30]. The deduced amino acid (aa) sequences of all six amplified genes were concatenated and aligned with those of the previously described LCDV species using the Mafft v7 program [32] with default parameters. Maximum likelihood (Phyml) program with 1000 sampling and Bayesian phylogenetic analyses were performed using MrBayes [33] within the TOPALi v2.5 program package [34] after removing the gaps from the alignment (2007 aa). Before the calculations, model selection was carried out in the program package, and for both methods, the JTT amino acid substitution model proved to be the best.

The program parameters for the Bayesian statistics were as follows: default prior probability distribution, the Markov chain was run for 10 million generations, and four independent analyses were conducted, each with one cold and three heated chains. Sampling occurred every 10 generations, with the first 25% of the Markov chain Monte Carlo samples discarded as burn-in.

### 2.4. Transmission Electron Microscopy

Transmission electron microscopy samples were collected only from the copperband butterflyfish. Immediately after collection, lymphocystis nodule samples were fully immersed in a freshly mixed (made within 24 h) solution of 0.2% glutaraldehyde in 4.0% buffered formalin. After two days of immersion fixation, the samples were transferred to a solution of pure 4% buffered formalin. The samples were refrigerated in the fixative solution after collection until embedding. The embedding protocol followed standard transmission electron microscopy (TEM) processing with a few notable modifications [35]. Embedding methods were lengthened due to the relatively broad width of the lymphocysts to allow for adequate dehydration and resin embedding. This allowed the preparation of whole lymphocytes and the maintenance of their cytostructural integrity. The standard embedding protocol was modified as follows: washing the samples in 0.1M phosphate buffer (PB) (10 min), pre-embedding contrast and fixation in 0.1% OsO_4_ (1 h), time in each step of dehydration alcohol series through to 50:50 absolute alcohol: propylene oxide (10 min), both propylene oxide steps (5 min), and 50:50 Durcupan resin: propylene oxide (2 h) Samples in freshly combined Durcupan resin were poured in silicon trays to form resin ‘bullets’. To facilitate the accessibility of ultramicrotome sectioning after the embedding process, the samples were carefully moved to the tip of the bullets while still fully encased in resin. The samples were kept under a hood at room temperature for 48 h and subsequently placed in a thermostat to cure for an additional 48 h. The lymphocyst nodules (fibroblast giant cells suspected to be infected with LCDV) were thereby retained whole and encased in the tips of resin bullets, fully surrounded by resin. The samples were sectioned using an ultramicrotome and subsequently fixed by post-embedding with lead citrate solution on mesh grids [35]. Samples were observed under a JEOL model JEM-1011 transmission electron microscope (JEOL Ltd., Tokyo, Japan) operating at 80 kV.

## 3. Results

**Figure 1:**
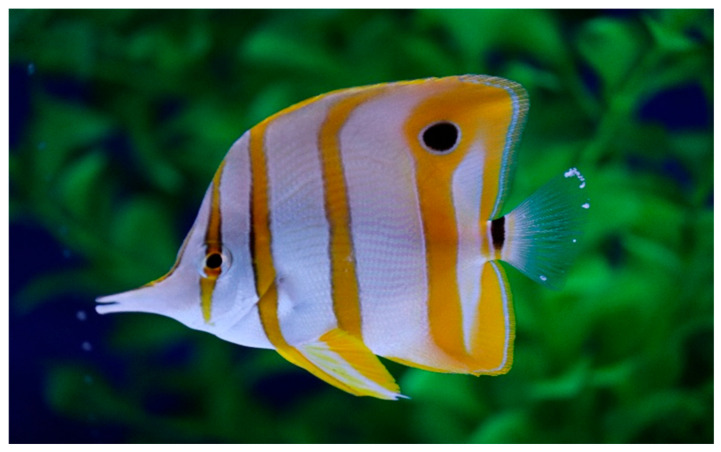
Cauliflower-like nodules on the external surface of the copperband butterflyfish (caudal fins).

**Figure 2:**
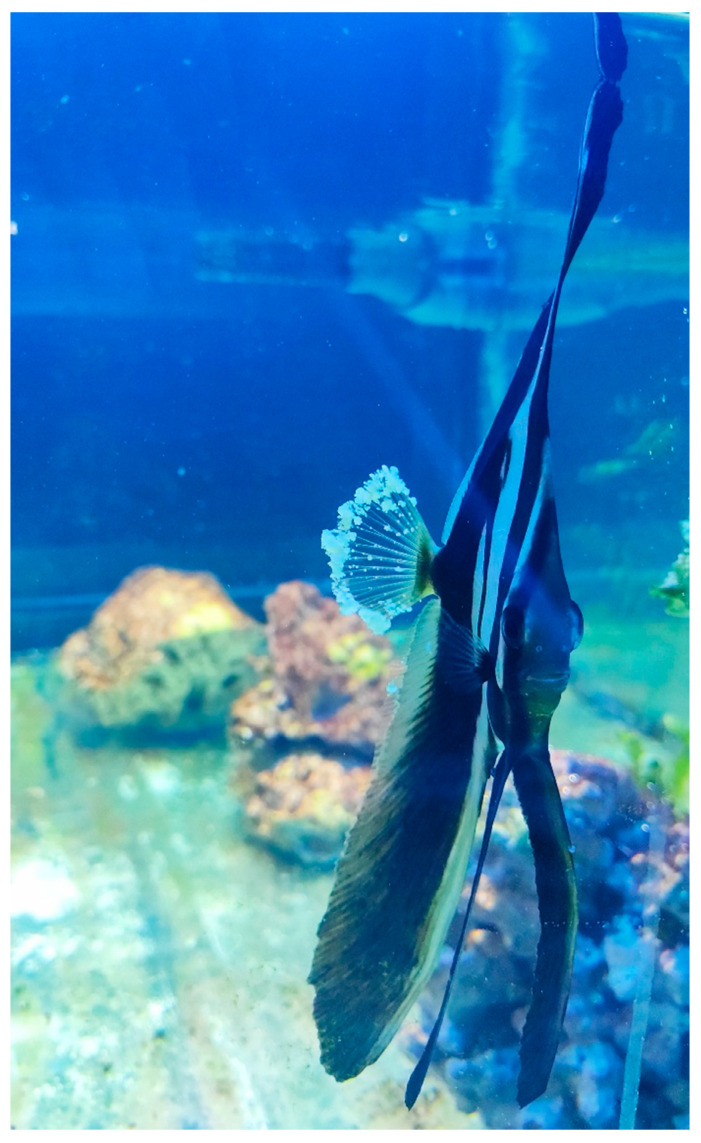
Whitish, cauliflower-like nodules on the caudal fins of the orbicular batfish.

**Figure 3:**
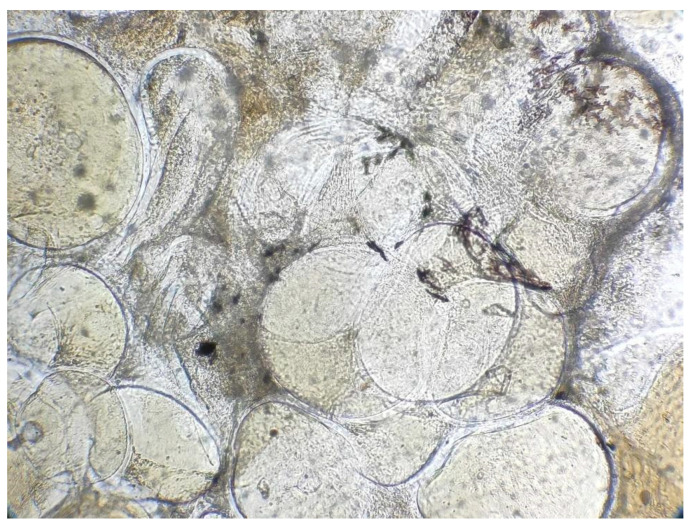
Native infected giant cells under light microscope from orbicular batfish (50X).

Infected animals showed whitish nodules (0.2–4.0 mm) on the external surface of their body and fins (Figure 1 and Figure 2). After collection, these nodules showed giant cells natively under light microscopy at 50X magnification (Figure 3).

### 3.1. Histopathology

**Figure 4:**
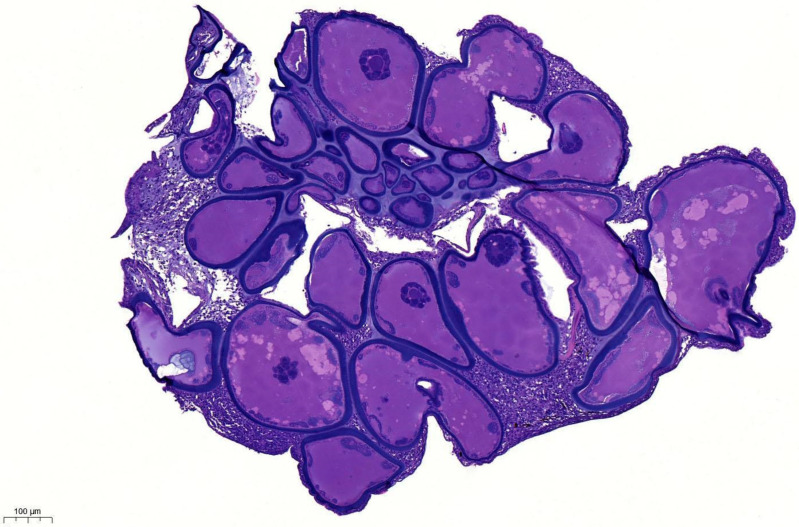
General view of lymphocystis nodule from copperband butterflyfish (H&E, Bar = 100 µm).

Histopathological examination revealed hypertrophied cells in a nodular mass (Figure 4).

**Figure 5:**
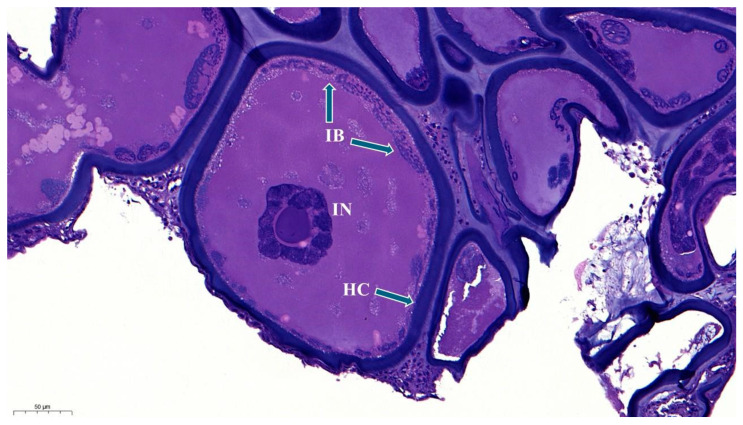
Lymphocystis nodule from the fin of the copperband butterflyfish, with a thick smooth hyaline capsule (HC), inclusion bodies (IB), and irregular nucleus (IN), (H&E. Bar = 50 µm).

A thick, smooth, hyaline capsule surrounded the malformed cells. Within a heterophilic cytoplasm, basophilic intracytoplasmic inclusion bodies were visible at the periphery of the hyaline capsule. The nuclei of the cells were irregular, enlarged, and contained basophilic marginated chromatin (Figure 5). Histology of both animals showed similar results.

### 3.2. Transmission Electron Microscopy of Copperband Butterflyfish

Qualitative TEM is clinically useful in presumptive LCD cases for detecting the presence of icosahedral virions, which strongly supports a diagnosis of LCDV infection. Additionally, the qualitative ultrastructural findings described below may indicate the first in vivo electron micrographs of LCDV particles in the copperband butterflyfish.

**Figure 6:**
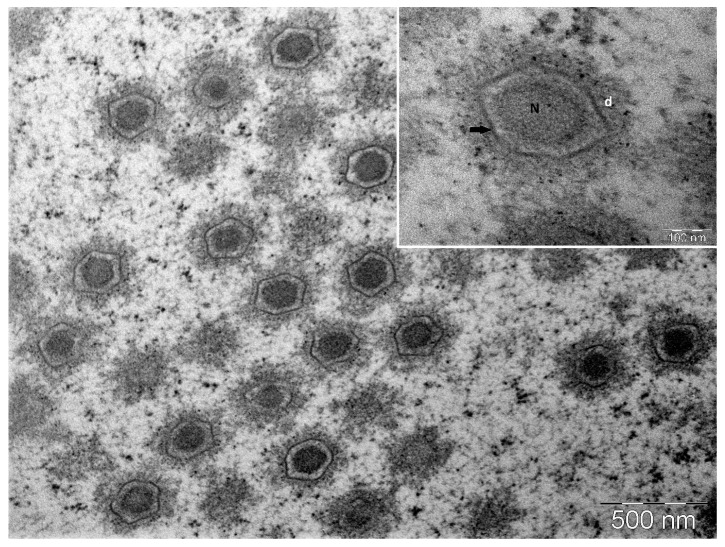
Transmission electron micrograph (TEM) of LCDV-infected giant cell at low (40kX) magnification showing loose lattice-like organisation of virions. Inset: TEM micrograph at high (200kX) magnification showing details of the virion. N: nucleoprotein core. Arrow: inner membrane. d: electron density associated with the inner membrane surrounding the virion. (Bar = 500 nm).

**Figure 7:**
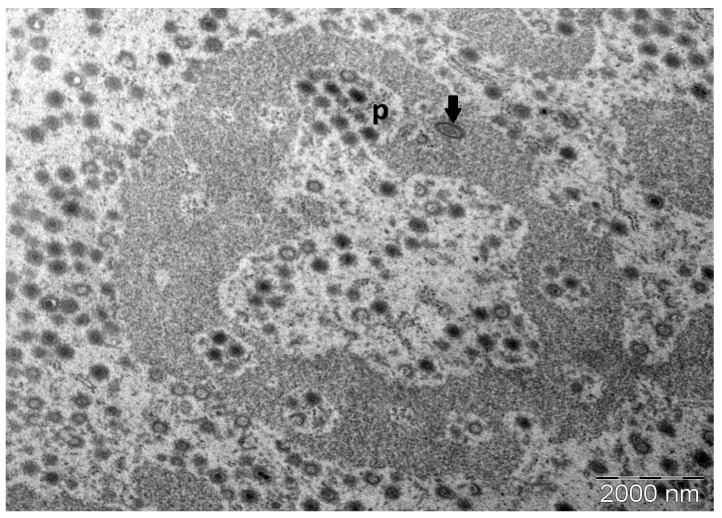
TEM micrograph of LCDV-infected giant cell showing intracytoplasmic inclusion body (ICIB) at low (10kX) magnification. Different compartments show different stages of viral assembly. p: paracrystalline array contained in ICIB. Arrow: virion with viral envelope (Bar = 2000 nm).

**Figure 8:**
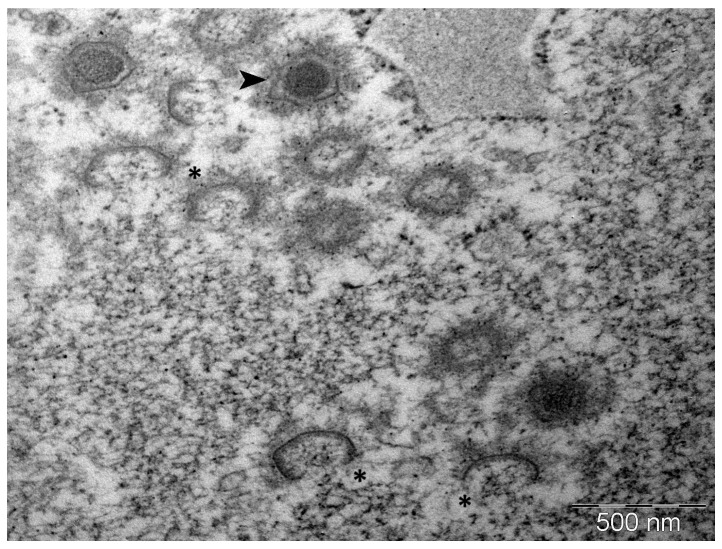
TEM electron micrograph of LCDV-infected giant cell ICIB at 50kX magnification, providing an intimate view of virion assembly. Arrowhead: Late-stage virion assembly with electron-dense nuclear core almost fully surrounded by inner membrane and associated capsomers. *: Early-stage virion assembly devoid of nuclear core densities with incomplete inner membranes. Note the marked difference in electron density surrounding the virions at different stages of assembly: in early stages, the circumscribed density is electron-lucent, while in late-stage assembly, the circumscribing density is more electron-dense. This difference in electron density may indicate a shift in inner membrane or capsomer-linked protein associations during virion assembly (Bar = 500 nm).

Virions in the lymphocyst sections were easily found under TEM at low magnification as the hypertrophic lymphocyst cells were filled to the brim with icosahedral virions, segregating the organelles and often the nucleus and the periphery. LCDV virions were observed in loose lattice-like aggregates (Figure 6) and true paracrystalline arrays (Figure 7). Intracytoplasmic inclusion bodies (ICIBs) showed signs of forming various compartments at different stages of viral assembly and unenveloped virion organisation (Figure 7). These different stages of virion assembly were also qualitatively associated with capsid circumscribing electron densities of varying structures and electron-lucence (Figure 8). These electron-dense areas were markedly more electron-lucent when found surrounding capsids in early-stage viral assembly. Additionally, these electron-dense areas were not observed in any of the enveloped viruses visualised within lymphocysts, either in ICIBs or in the lymphocyst cytoplasm (Figure 7). These electron densities were observed when virions were loosely organised in a lattice-like structure; however, when unenveloped virions were seen in paracrystalline arrays, these circumferential electron densities were notably not apparent. These electron densities surrounding the viral capsids may indicate protein machinery associated with cytoskeletal organisation of virions and protein assemblages associated with LCDV virion capsomeres or intramembraneous proteins.

### 3.3. Results from PCR Assay and Phylogenetic Analysis of Orbicular Batfish

All PCRs targeting the DNA from the paraffin-embedded tissue originating from the copperband butterflyfish failed. The total DNA concentration was very low (>0.5 ng/µL). After the non-specific DNA amplification, the PCRs were repeated, but were again unsuccessful.

**Figure 9:**
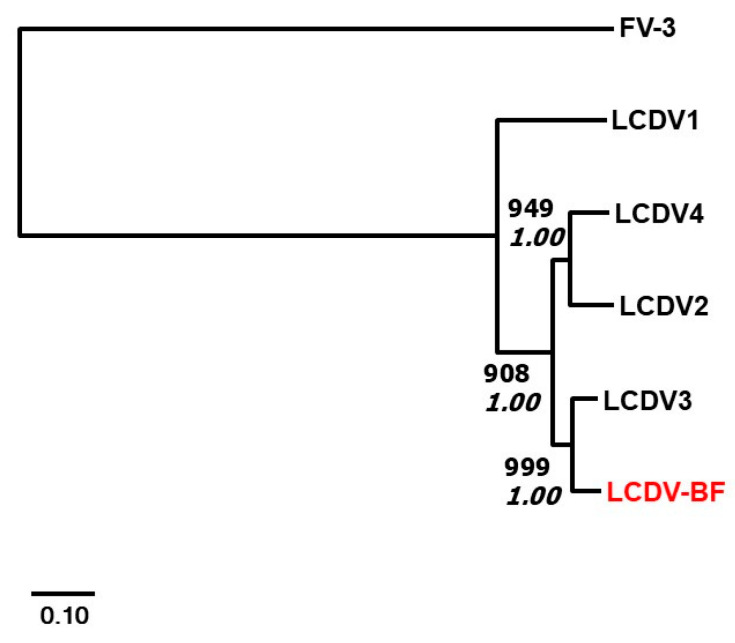
Phylogenetic relationships within the four members of the genus *Lymphocystivirus* and the novel virus: batfish lymphocystis disease virus (LCDV-BF, marked with red color). Analyses were based on the Bayesian analysis and ML method (JTT amino acid substitution model for both methods) of the concatenated deduced amino acid sequences (2007 aa) of six genes (DNA polymerase, RNA polymerase, MCP, ATPase, core genes 9, and 20). The trees show that batfish LCDV clusters into the genus *Lymphocystivirus* and show a clear separation of the novel virus from the other LCDV species. High statistical values (bootstrap values for ML in bold and inference support for Bayesian analysis in italics) confirm the topology of the trees. Abbreviations: LCDV: lymphocystis disease virus; FV-3: frog virus 3.

PCRs from the batfish sample produced a 374 bp DNA fragment from the DNA polymerase gene and a 1303 bp region from the MCP gene. Subsequently, from the ATPase (core gene 21), core gene 9, core gene 20, and RNA pol (core gene 2), 733, 463, 1131, and 2077 bp fragments were amplified, respectively. The sequences were deposited in GenBank (Acc. No.: PV891874-79). The G + C content of the concatenated nucleotide sequences of the novel LCDV (LCDV-BF) was 41.75%. The predicted protein identity of the amplified genes to their counterparts in the genomes of the four accepted LCDV species is shown in Table 1. Figure 9 shows the phylogenetic relationships within the genus *Lymphocystivirus,* containing the officially accepted four members of the genus and the novel virus, proposed as batfish lymphocystis disease virus (LCDV-BF) and a ranavirus (Frog virus 3, FV-3) as an outgroup. Both Bayesian and ML methods resulted in the same tree topology supported by high statistical values. According to calculations, LCDV-BF could be classified as a sister species of LCDV-3 within the genus *Lymphocystivirus*.

## 4. Discussion

This is the first report of a novel batfish lymphocystis disease virus (LCDV-BF) found in the orbicular batfish. The macroscopic and microscopic findings were similar to the common characteristics of LCDV infection in other fish species [21,36]. The organisation of the virions intracellularly and the ultrastructure of the virions in different stages of assembly were compared primarily to the LCDV descriptions in Fenner’s Virology [37].

Histology and electron microscopy were performed on the copperband butterflyfish, confirming LCDV qualitatively from gross to ultrastructural levels. Performing PCR and phylogenetic analyses of the copperband butterflyfish samples were not successful because the isolates from paraffin-embedded sections were too weak for amplification. In the orbicular batfish case, PCR and phylogenetic analyses were successfully performed, supporting a separate putatively novel LCDV species. LCD is hereby described following stressful management events in two novel ornamental fish species, qualitatively in the copperband butterflyfish by electron microscopy and quantitatively in the orbicular batfish by PCR. In addition, following phylogenetic analyses of the LCDV isolated from the orbicular batfish, a putatively novel LCDV was proposed.

Qualitative TEM in this case of LCDV infection offers rare insights into viral assembly within ICIBs of lymphocysts. Rigorous quantitative TEM is neccesary to untangle the precise protein associations of virion assembly and assemblage in paracrystalline arrays. The formation of these assemblages and the extent of their interaction with host cytoskeletal architectural systems may also be elucidated by quantitative TEM in the future. Additionally, without rigorous quantitative TEM analyses of lymphocyst-infected cell cytoskeletal architecture and capsid-associated protein associations of LCDV, it is impossible to say for certain what the observed capsid circumscribing electron densities may be. Here, we merely show an increase in electron-density associated with late-stage compared to early-stage LCDV virion assembly. We hypothesise that this increase in circumferential electron density may be associated with end-stage icosahedral capsule formation and/or may be associated with the organisation of the virions into paracrystalline arrays within the cytoplasm of lymphocysts.

Viral diseases pose a significant challenge to aquaria housing marine teleost species. While LCDV manifests in cases of immune suppression, it can pose an infectious threat and infect individuals horizontally. *Artemia* spp. may play an important role in the infection as host and vector species [38]. While there must be a general dip in the immunity of species affected by lymphocystis disease, it may spread through aquarium collections through infectious processes with concurrent overarching immunocompromise, often secondary to suboptimal management. No vaccines are available against any LCDV [5,39].

However, until a vaccine is available, prevention is key to avoiding lymphocystis disease. As with all infectious diseases, a robust quarantine system is of utmost importance. Quarantine systems should include separate filtration and disinfection systems (UV, ozone) and should be in place for an adequate amount of time.

Known iridovirus genomes, including that of the LCDV, share 26 core genes, of which six were partially amplified and sequenced from LCDV-BF. Within the family Iridoviridae, the species demarcation criteria of the International Committee on Taxonomy of Viruses (ICTV) are as follows: viruses sharing 95% or greater amino acid identity (26 core genes) are considered to belong to the same virus species. Moreover, they must have similar genome sizes and G+C contents, and should show phylogenetic relatedness and co-linear gene arrangement (https://ictv.global/files/proposals/approved?page=22, document: 2018.007D.A.v1.Iridoviridae_8sp3sprem.docx (accessed on 26 September 2025)). Only the MCP showed amino acid similarity greater than 95% to their counterparts in the accepted LCDV species (Table 1). Interestingly, it showed greater than 95% identity to two LCDVs, 95.4% identity to LCDV-2, and 97.7% identity to LCDV-3. Phylogenetic calculations based on the concatenated sequence of six partially sequenced core genes show that LCDV-BF clusters into the genus Lymphocystivirus, and the separation of LCDV-BF from the other LCDVs is significant (Figure 6). Due to horizontal gene transfer and variable rates of evolution, all core genes need to be sequenced before the proper evolutionary position/status of LCDV-BF within the genus can be determined [40]. However, in a previous study, an LCDV originating from whitemouth croaker (*Micropogonias furnieri*) was described and characterised using the same six partial gene sequences, and the virus was tentatively considered a novel species, LCDV [30]. Later, complete genome sequence analysis of the virus proved that it was a novel species (LCDV-4) within the genus *Lymphcystivirus* [41], suggesting that complete genome sequences are not always necessary to establish a novel species. Taking into account these findings, the authors consider LCDV-BF as a tentatively novel LCDV species closely related to LCDV-3.

## Figures and Tables

**Table 1:** Predicted amino acid sequence comparison of six partially sequenced genes of LCDV-BF with their counterparts in the four recognised LCDV species.

LCDV-BF	LCDV-1	LCDV-2	LCDV-3	LCDV-4
MCP (451 aa)	88.22%	95.38%	97.69%	93.76%
ATPase (244 aa)	61.07%	87.30%	93.03%	85.25%
DNApol (139 aa)	52.38%	84.68%	88.71%	85.48%
RNA pol (692 aa)	67.58%	81.79%	91.91%	83.24%
Core gene 20 (376 aa)	68.09%	88.56%	93.09%	90.69%
Core gene 9 (154 aa)	76.62%	91.56%	94.16%	93.51%

## Data Availability

The data presented in this study are available upon request from the corresponding author.

## Abbreviations

The following abbreviations are used in this manuscript:

LCD	lymphocystis disease
LCDV	lymphocystis disease virus
LCDV-BF	batfish lymphocystis disease virus
TEM	transmission electron microscopy
ICIB	intracytoplasmic inclusion body
H&E	haematoxylin–eosin
KH	carbonite hardness
NO_3_^−^	nitrate ion
Ca	calcium
Mg	magnesium
